# CPAP Therapy on Depressive and Anxiety Symptoms in Patients with Moderate to Severe Obstructive Sleep Apnea Syndrome

**DOI:** 10.3390/medicina58101402

**Published:** 2022-10-06

**Authors:** Diana Raluca Velescu, Monica Marc, Diana Manolescu, Daniel Trăilă, Cristian Oancea

**Affiliations:** 1 Center for Research and Innovation in Precision Medicine of Respiratory Diseases, ”Victor Babes” University of Medicine and Pharmacy Timisoara, Eftimie Murgu Square 2, 300041 Timisoara, Romania; 2 Department of Radiology, “Victor Babes” University of Medicine and Pharmacy Timisoara, Eftimie Murgu Square 2, 300041 Timisoara, Romania

**Keywords:** depression, anxiety, obstructive sleep apnea, continuous positive airway pressure

## Abstract

*Backgrounds and Objectives:* There is a link between sleep apnea syndrome (OSA) and depressive and anxiety symptoms, but the underlying mechanisms are not fully understood. The study aimed to determine the prevalence of these symptoms in patients with OSA and to evaluate the impact of continuous positive airway pressure (CPAP) on depression and anxiety scores. *Materials and Methods:* Ninety-nine consecutive new patients diagnosed with moderate or severe (apnea-hypopnea index AHI ≥ 15 events/h) OSA were accepted for the CPAP therapy. Patients completed a patient health questionnaire (PHQ-9) for depressive symptoms and a generalized anxiety questionnaire (GAD-7) for anxiety symptoms before the CPAP treatment, after 6 months, and after 1 year of CPAP therapy with compliance. For both scores (PHQ-9 and GAD-7), a cut point ≥10 was used to indicate the presence of clinically depressive and anxiety symptoms. *Results:* Forty-eight individuals (48.48%) had PHQ-9 scores above the cut-off point ≥ 10, and twenty-seven participants (27.27%) had GAD-7 scores above the cut-off point ≥10. A significant correlation has been shown between PHQ-9 score and BMI (*p* < 0.0001), AHI (*p* < 0.0001), ODI (*p* < 0.001), ESS (*p* < 0.001), GAD-7 score (*p* < 0.0001), and [t90] (*p* < 0.0001), while GAD-7 was correlated with AHI (*p* < 0.030), ODI (*p* < 0.006), and [t90] (*p* < 0.001). The PHQ-9 and GAD-7 scores decreased significantly after 6 months of CPAP therapy in all groups and after 1 year of CPAP use. *Conclusions:* OSA patients should be screened for depression and anxiety. Furthermore, CPAP should be the first choice of treatment before starting other treatments for depression and anxiety symptoms.

## 1. Introduction

Patients with obstructive sleep apnea (OSA) manifest similar symptoms to those with depression and anxiety. Although the mechanism underlying this relationship is not fully understood, current studies have shown that the prevalence of symptoms of depression and anxiety in patients with untreated OSA is higher [1,2]. A 2019 meta-analysis that involved 73 articles estimated that the prevalence of depressive symptoms and anxiety in a study population with OSA are up to 35% and 32%, respectively [1]. Symptoms of OSA include snoring, apnea reported by the entourage, choking episodes, sleep fragmentation, insomnia, nocturia, morning headaches, excessive daytime sleepiness, irritability, altered concentration, decrease in memory, and loss of libido [3].

Studies showed that the prevalence of moderate-to-severe sleep-disorder breathing in adults is 23.4% in women and 49.7% in men [4]. OSA is an independent risk factor for cardiovascular diseases, essential hypertension [5,6], coronary artery disease, stroke, cerebrovascular disease [7,8], metabolic syndrome [9], and diabetes [10]. Sleep fragmentation, hypoxia, and persistent sleep loss lead to symptoms, such as depression since the frontal lobe centers responsible for emotions are sensitive to poor quality sleep [11]. Moreover, patients with clinical depression present anxiety symptoms expressed by psychological and physical distress and irritability [12]. Understanding the importance of the relationship between these psychiatric comorbidities and OSA might improve the diagnostic and treatment outcomes for both [13].

The standard gold treatment for OSA is represented by continuous positive air pressure (CPAP), which prevents upper airway collapse during sleep. Some studies from the literature reported a positive effect on depressive and anxiety symptoms, while others showed inconstant findings [13,14]. In addition, different scales for diagnosing depression and anxiety with different criteria and cut-off points may lead to variation in reported conclusions in different studies [15].

The present study aimed to determine the prevalence of depressive and anxiety symptoms in OSA patients and to study the effect of CPAP therapy on these psychological conditions over 1 year of follow-up.

## 2. Materials and Methods

### 2.1. Subjects

Patients were recruited from the sleep laboratory of the Clinical Hospital of Infectious Diseases and Pneumophtisiology ‘‘Dr. Victor Babes” Timisoara. Those who were found to have moderate or severe OSA (AHI ≥ 15 events/h) and agreed to use CPAP therapy for 1 year by giving informed consent were included in the study. Patients with previous psychiatric diagnoses or who were under psychiatric medication were excluded from the study. Moreover, the exclusion criteria were alcohol consumption, serious or unstable associated conditions with chronic disease, and those who were unable to complete the questionnaires meaningfully.

The ethics board approved the study of the Clinical Hospital of Infectious Diseases and Pneumophtisiology ‘‘Dr. Victor Babes” Timisoara. Participants of the study accepted to sign the written informed consent.

### 2.2. Measurements

Demographic data, smoking status, and comorbidities were collected from all included patients.

All patients underwent overnight cardiorespiratory polygraphy using the Sleep Doc Porti 7 system, Version 5.19b, Hechingen, Germany, which included oximetry to measure oxygen desaturation, a thermistor sensor for breathing, a thoracic effort belt for respiratory movements, and a microphone for snoring. According to the American Academy of Sleep Medicine “Chicago” from 1999 criteria, apnea was defined as a cessation of airflow for 10 s, and hypopnea was defined as a decrease in amplitude with 50 to 90% airflow reduction from baseline, associated with >3% oxygen desaturation or arousal. Moderate to severe OSA was defined as an Apnea Hypopnea Index ≥15 events/h. The amount of time spent during sleep with an arterial oxygen saturation level below 90% indicated hypoxemia [t90]. The patients were instructed to avoid alcohol, medicines affecting sleep, and sleeping during the daytime before performing polygraphy.

### 2.3. Self-Reported Measures

Eligible OSA patients were assessed using three tools:

- Epworth Sleepiness Scale (ESS) was used to assess daytime sleepiness. Patients were asked to indicate the tendency to fall asleep in 8 passive or active specific situations on a 0–3 scale, with 0 meaning no chance of falling asleep and 3 showing a high chance of falling asleep. A score of 10 means excessive daytime sleepiness [16].
- Patient Health Questionnaire-9 Depression Scale is a self-reported validated questionnaire that is used to assess the presence and the severity of depression by primary care practitioners [17]. It can establish depressive distress as well as the grade of depressive symptoms severity. The scale consists of 9 items that assess the patient health status during the previous 2 weeks and relate to feelings of sadness, tiredness, sleepiness or sleeping too much little interest in doing things, thoughts of personal failure, poor concentration, low self-confidence, slow or fast speech, and suicidal ideation. From the maximum score of 27 points, a cut-off value ≥ 10 was used to indicate the presence of clinically significant depressive symptoms [18].
- Generalized Anxiety Disorder Questionnaire-7 Scale is a validated questionnaire that measures the severity of anxiety. Seven questions refer to feeling nervous, on edge, unable to control worrying, being worried about different things, inability to relaxbeing very restless that it is hard to sit, getting irritated easily, and feeling afraid that something horrible might happen. A total score ranges from 0 to 21, and the cut-off point for moderate anxiety symptoms is over 10 [19].

Patients were assessed at baseline immediately after the sleep study and confirmation of moderate or severe OSA, a second evaluation was completed after 6 months of CPAP therapy, and a third one was conducted 1 year after initialization of CPAP treatment. At baseline and for each evaluation, we asked patients to complete ESS, PHQ-9, and GAD-7 scales. A psychiatrist evaluated each patient at the baseline to establish if the patients could finish the study without specific psychiatric medications.

All CPAP-compliant participants (mean use from device download ≥ 5 h/night) were reassessed for depressive symptoms with the PHQ-9 scale and anxiety with the GAD-7 scale after 6 months of usage and 1 year of treatment.

### 2.4. Statistical Analysis

Data were collected and analyzed using SPSS version 26 (SPSS Inc, Chicago, IL, USA) and are presented as medians and interquartile ranges for continuous variables without Gaussian distribution or percentages for categorical variables. To assess the significance of the differences between groups, the Mann–Whitney U test, Kruskal–Wallis (medians, non-Gaussian populations), and χ2 (proportions) tests were used. Continuous-variable distributions were tested for normality using the Shapiro–Wilk test and for equality of variance using Levene’s test. The strength of association between two continuous variables from non-Gaussian populations was evaluated using Spearman’s correlation coefficient. The sample size calculation was performed prior to the study, aiming to provide a confidence level of 95%. In this study, *p* < 0.05 was considered the threshold for statistical significance.

## 3. Results

The study comprised 147 participants, and 48 were excluded according to our study criteria. Ninety-nine consecutive patients diagnosed with moderate to severe OSA, according to AHI, were eligible for this study and accepted the CPAP treatment for no less than 1 year. Sixty-six men and thirty-three women provided informed consent and were included in the study. The mean age of participants was 56.49 ± 10.92; BMI 36.54 ± 6.4 (kg/m^2^); neck circumference 44.08 ± 3.82; abdomen circumference 124.67 ± 14.47, with no significant difference between men and women. Table 1 shows the characteristics of the study group at baseline. Forty-eight individuals (48.48%) had PHQ-9 scores above the cut-off point ≥ 10, and twenty-seven participants (27.27%) had GAD-7 scores above the cut-off point ≥10 (Table 1).

Spearman’s test showed a correlation between PHQ-9 score and BMI (*p* < 0.0001, r = 0.40), AHI (*p* < 0.0001, r = 0.40), ODI (*p* < 0.001, r = 0.47), ESS (*p* < 0.001, r = 0.47), GAD-7 score (*p* < 0.0001, r = 0.58), and [t90] (*p* < 0.001, r = 0.53). Correlation analyses of the GAD-7 score showed that GAD-7 was correlated with AHI (*p* < 0.030, r = 0.20), ODI (*p* < 0.006, r = 0.27), and [t90] (*p* < 0.001, r = 0.25).

**Figure 1:**
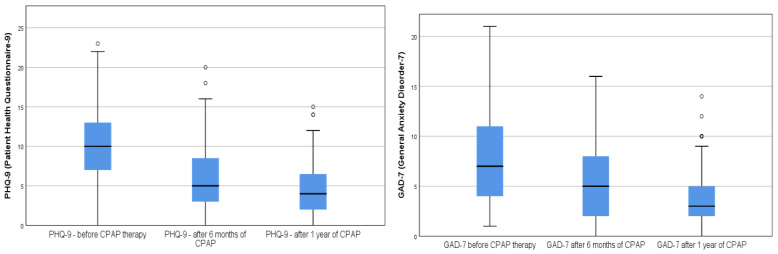
Boxplot showing the difference in PHQ-9 and GAD-7 progression following CPAP therapy. PHQ-9, patient health questionnaire; GAD-7, generalized anxiety disorder questionnaire. The circles represent the outliners of the boxplot.

OSA patients had a high risk of associated comorbidities. These were in order of frequency: Essential hypertension (63%), ischemic heart disease (28%), diabetes mellitus (22%), COPD (16%), heart failure (14%), asthma (6%), arrhythmia (8.1%), stroke (2%). The patients with more comorbidities tend to be more anxious; the GAD-7 score correlates with the sum of comorbidities (*p* < 0.03). The PHQ-9 and GAD-7 scores decreased significantly after 6 months of CPAP therapy in all groups and after 1 year of CPAP use (Table 2). Of the forty-eight patients with clinically significant depression (PHQ-9 ≥ 10) at the end of the study, only seven individuals (two men and five women) met the diagnosis of moderate clinical depression (7.07%). In the group of patients with clinical anxiety (GAD-7 ≥ 10), 27.27% decreased to 2.02% after 1 year of CPAP therapy. The PHQ-9 score decreased after 6 months of therapy from 10 (3–7) to 5 (3–8.5), respectively at 1 year, 4 (2–6.5). Spearman’s test showed a strong correlation between PHQ-9 after 6 months of therapy (r = 0.81, *p* < 0.001), respectively at 1 year (r = 0.92, *p* < 0.001). Moreover, the GAD-7 score has a strong correlation at 6 months of therapy (r = 0.94 *p* < 0.001 and after 1 year of CPAP therapy (r = 0.84, *p* < 0.001) (Figure 1).

**Figure 2:**
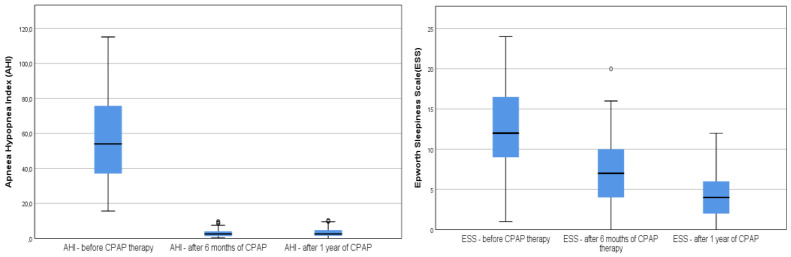
Boxplot showing the difference inAHI and ESS progression following CPAP therapy. AHI, apnea hypopnea index; ESS, epworth sleepiness scale. The circles represent the outliners of the boxplot.

AHI decreased after 6 months of therapy from 54 (37.05–75.75) to 2.5 (1.5–4.7), respectively at 1 year, 2.5 (1.3–6.4) (Figure 2). Spearman’s test showed a weak correlation between AHI after 6 months of therapy (r = 0.37, *p* < 0.001), respectively and a strong correlation after 1 year (r = 0.64, *p* < 0.001). Moreover, ESS has a strong correlation at 6 months of therapy (r = 0.81, *p* < 0.001), and after 1 year of CPAP therapy (r = 0.79, *p* < 0.001) (Figure 2).

## 4. Discussion

This study was aimed to determine the prevalence of depressive and anxious symptoms in moderate to severe OSA patients, the relation between different variables of sleep apnea with depression and anxiety, as well as the effect of CPAP treatment on the psychological well-being of study patients. During the study period, the number of male patients with OSA was twice the number of females (66 males and 33 females). This percentage might show the increased incidence of OSA in males reported in different studies [1,20]. For our research, we aimed to study the association between depression and anxiety with OSA. More than half of those studied participants reported depressive symptoms (51.52%), and forty-eight of study participants had clinically significant moderate to severe symptoms (48.48%). According to the PHQ-9 total score, females showed a higher score of depression than males, but this initial examination of the scale by gender reveals no statistically significant difference (*p* < 0.462). This result has been reported in previous studies [21]. Clinically significant depressive symptoms (PHQ-9 ≥ 10) were more common in men, but this finding can be explained by the double number of men included in the current study. The main difference between males and females could be in the degree of severity of depressive symptoms compared with the percentage of depression in OSA patients.

Disturbed sleep, fatigue, and irritability are symptoms associated with depression, anxiety, and OSA, but the association between these diseases is still poorly understood. Sleep fragmentation and oxygen desaturation could have a pathogenic role in the occurrence of depression [21]. Our analyses demonstrate a strong correlation with the severity of hypoxic events using AHI, an index of fragmentation, and [t90], an index of hypoxemia, with a PHQ-9 score in the untreated state. Sleep fragmentation represents a cause of excessive daytime sleepiness in OSA patients and is suggested to be found in depressive symptomatology [22,23]. In our findings, the correlation between the ESS scale and PHQ-9 was significant (*p* < 0.001) with r = 0.47.

Obesity itself was a predictive of depressive symptoms, with more obese patients scoring higher on the PHQ-9 score. ODI was related to depression (*p* < 0.0001) and obesity (*p* < 0.0001) in our study, with patients having a greater tendency toward obesity and approval of depressive symptomatology. It might be a possibility that severe oxygen desaturation during sleep plays an essential role in the development of neuropsychological disturbance, increasing the PHQ-9 score. The relationships between ODI obesity and depression have all been reported in other studies from the literature [24,25].

Moreover, OSA and depression have common risk factors associated with cardiovascular disease and metabolic syndrome [6,10]. The frequency of comorbidities in our study was represented by essential hypertension (63%), ischemic heart disease (28%), and diabetes mellitus (22%), as this result supports the previous argument.

The percentage of anxiety symptoms was higher (72.73%), and twenty-seven participants (27.27%) scored over 10 points. In our study, there was no significant difference between males and females, even if the total score of GAD-7 was higher in females (8.84) than in males (7.54). These data were shown in previous studies [2,26]. The high prevalence of anxiety symptoms is associated with the sum of comorbidities for the study population. In our study group, 55% of individuals were associated with at least one comorbidity, which strongly supported this hypothesis. The dates of the presence of anxiety in OSA are less common than depression but not unusual. Since we have demonstrated a high prevalence of anxiety in addition to the strong positive correlation between the two psychiatric comorbidities in our study population (*p* < 0.000), this might be a sight for future investigation into the relationship between OSA and mental health.

At follow-up, after 6 months of CPAP usage, a marked improvement in depression was found, as reflected by a statistically significant improvement in the PHQ-9 score (*p* < 0.001) and after 1 year of CPAP therapy (*p* < 0.027). A meta-analysis of 19 studies of the effective treatment of obstructive sleep apnea on depressive symptoms found that overall, there was an improvement in depressive symptoms, despite significant heterogeneity among the trials and outcomes [27]. In the current study, only seven patients (two men and five females) showed a total PHQ-9 total score higher than 10 points at the end of the study. These patients were referred to a psychiatrist for medical treatment and CPAP therapy. PHQ-9 score was previously demonstrated to be a responsive measure of depressive symptoms using CPAP treatment in OSA. This effect was shown after 3 months of CPAP adherence using 5 h of CPAP therapy [20].

Regarding the results of our study, we identified a decrease in GAD-7 total score at follow-up (from 7.98 ± 5.05 to 5.54 ± 3.84 after 6 months of CPAP and to 3.78 ± 3.16 after 1 year of CPAP). Only nine participants (three males and six females) in the study manifested anxiety symptoms (GAD-7 ≥ 10) clinically. Each of these was associated with at least one comorbidity. A recent study that investigated the benefits of CPAP therapy on mood alteration, sleepiness, and weight concluded that long-term CPAP therapy improved anxiety symptoms using a toll State-Trait Anxiety Inventory (STAI) questionnaire [28]. These results indicate a benefice effect of CPAP therapy on depressive and anxiety symptoms.

OSA severity measured by AHI showed a strong significant correlation both in the PHQ-9 score (*p* < 0.000) and in the GAD-7 score (*p* < 0.030). As we have seen in the current study, CPAP therapy improved the mean AHI due to a follow-up period of 6 months from 56.45 ± 24.23 to 3.43 ± 2.62, and after 1 year, to 3.07 ± 2.16. Moreover, CPAP improved excessive daytime sleepiness over 1 year of treatment from 12.42 ± 5.12 to 4.45 ± 2.86. In addition, by reducing respiratory events with CPAP therapy, symptoms of depression and anxiety improved, as well.

Despite the benefits of CPAP therapy on excessive daytime sleepiness, mood alteration, depression, anxiety, and ability to concentrate, patients experience a burden in using it, which is similar to financial, social, and psychological problems [29]. In many countries, the government does not sustain the cost of therapy, and patients can not afford to start the treatment. Studies showed that untreated OSA is associated with significant financial pressure on health care. Moreover, productivity, workplace incidents, car accidents, and comorbidities increase the estimated costs. Joshua M. Bock et al. concluded that the annual care cost of adherent CPAP patients with cardiovascular comorbidities was lower than those of the non-adherent group (USD 6825, USD 11,312; *p* < 0.05), and health care investment was reduced by 40% in 1 year [30].

Recent studies demonstrated that adherence to CPAP therapy is not the only factor for successful treatment; lifestyle behavior, physical activity, and a healthy diet improve its effects [31]. Alcohol consumption and smoking may play an essential role in OSA by increasing sleep disruption through pharyngeal resistance and airway inflammation [32,33]. Therefore, smoking cessation and control of alcohol drinking prevent the risk of airway collapsibility during sleep. Intense physical activity has a contributory role in losing weight, but it also decreases the level of blood pressure and biomarkers of inflammation [34]. A study by Reid et al. found that a diet rich in red/processed meat and lower whole grain consumption is associated with moderate to severe OSA by a diminution in slow-wave sleep, which increases sleep fragmentation [35].

The study has its limitations. The number of participants in the study relates to the unequal number of males and females. As we know, women are more vulnerable to mood alteration. The study used a single score to evaluate depressive symptoms. However, it is acknowledged that the PHQ-9 is a valid questionnaire based on one of the nine DMS-IV depression criteria, but it is unknown whether depression is a primary consequence of OSA or a secondary result of OSA-related symptoms, such as excessive sleepiness, fatigue, sleep problems or irritability. Even if we have improved the total PHQ-9 score by individual questions from the scale during follow-up, a recent study highlights the need for studies using scales that do not include somatic symptoms associated with OSA [2]. Using a questionnaire that does not include items about sleepiness and fatigue, such as HADS-D (Hospital Anxiety and Depression Scale) may be reliable for identifying depressive symptoms. Nevertheless, a study concluded that the score improved with CPAP therapy only in adherent group patients (≥4 h/night) [14].

## 5. Conclusions

In conclusion, depression and anxiety were prevalent in moderate to severe OSA patients and were more severe in women. However, improving OSA with CPAP therapy decreased the severity of depressive and anxiety symptoms. Therefore, it is important to avoid the unnecessary use of psychiatric medication since a large group of patients will improve clinically using CPAP therapy.

## Figures and Tables

**Table 1:** Description of a study group at baseline.

	Total (n = 99)	Male (n = 66)	Female (n = 33)	*p-Value*
Age	56 (48, 65)	55 (44, 63)	62 (54, 68)	<0.001
BMI	36 (31, 40)	36 (31, 40)	37 (34, 41)	0.24
Neck (cm)	44.0 (41.0, 47.0)	44.5 (41.2, 47.8)	43.0 (40.0, 46.0)	0.041
Abdomen (cm)	127 (112, 136)	130 (114, 136)	122 (109, 134)	0.14
AHI	54 (37, 76)	54 (35, 77)	54 (39, 73)	0.92
ODI	54 (30, 71)	55 (29, 73)	52 (33, 58)	0.64
[t90]	21 (4, 46)	22 (4, 56)	20 (4, 39)	0.68
ESS	12.0 (9.0, 16.5)	8.0 (3.2, 10.0)	6.0 (4.0, 8.0)	0.48
PHQ-9 score	10.0 (7.0, 13.0)	9.5 (7.0, 12.0)	12.0 (10.0, 15.0)	<0.001
PHQ-9 ≥10 n (%)	48 (48.48)	39 (39.39)	12 (12.12)	0.033
GAD-7 score	7.0 (4.0, 11.0)	7.0 (3.0, 10.0)	7.0 (5.0, 12.0)	0.25
GAD-7 ≥10 n (%)	27 (27.22)	16 (16.16)	11 (11.11)	0.343

Results are presented as median and interquartile range; BMI, body mass index; AHI, apnea-hypopnea index; ODI, oxygen desaturation index; [t90], total sleep time spent at an arterial oxygen saturation <90%; ESS, Epworth sleepiness scale; PHQ-9, patient health questionnaire; GAD-7, generalized anxiety disorder questionnaire.

**Table 2:** Clinical depression and anxiety measured by PHQ-9 and GAD-7 before initiating CPAP therapy, after 6 months and 1 year of treatment.

	Total (n= 99)	Men (n = 66)	Women (n = 33)	*p*-Value
Baseline				
PHQ-9 score <10	51.0 (51.5%)	39.0 (59.1%)	12.0 (36.4%)	0.033 ^1^
PHQ-9 score ≥ 10	48.0 (48.5%)	27.0 (40.9%)	21.0 (63.6%)	
GAD-7 score < 10	72.0 (72.7%)	50.0 (75.8 %)	22.0 (66.7%)	0.338 ^1^
GAD-7 score ≥ 10	27.0 (27.3%)	16.0 (24.2%)	11.0 (33.3%)	
After 6 months				<0.001 ^1^
PHQ-9 score < 10	87.0 (87.9%)	63.0 (95.5%)	24.0 (72.7%)	
PHQ-9 score ≥ 10	12.0 (12.1%)	3.0 (4.5%)	9.0 (27.3%)	
GAD-7 score < 10	88.0 (89.9%)	60.0 (90.9%)	28.0 (84.8%)	0.366 ^1^
GAD-7 score ≥ 10	11.0 (11.1%)	6.0 (9.1%)	5.0 (15.2%)	
After 1 year				0.027 ^1^
PHQ-9 score < 10	92.0 (92.9%)	64.0 (97.0%)	28.0 (84.8%)	
PHQ-9 score ≥ 10	7.0 (7.1%)	2.0 (3.0%)	5.0 (15.2%)	
GAD-7 score < 10	97.0 (98.0%)	65.0 (98.5%)	32.0 (97.0%)	0.631 ^1^
GAD-7 score ≥ 10	2.0 (2.0%)	1.0 (1.5%)	1.0 (3.0%)	

PHQ-9, patient health questionnaire; GAD-7, generalized anxiety disorder questionnaire; ^1^ Chi-square test.
